# Validity of Anti-PSMA ScFvD2B as a Theranostic Tool: A Narrative-Focused Review

**DOI:** 10.3390/biomedicines9121870

**Published:** 2021-12-10

**Authors:** Barbara Frigerio, Elena Luison, Alessandro Desideri, Federico Iacovelli, Chiara Camisaschi, Ettore C. Seregni, Silvana Canevari, Mariangela Figini

**Affiliations:** 1 Biomarkers Unit, Department of Applied Research and Technical Development, Fondazione IRCCS Istituto Nazionale dei Tumori, 20133 Milan, Italy; barbara.frigerio@istitutotumori.mi.it (B.F.); elena.luison@istitutotumori.mi.it (E.L.); chiara.camisaschi@istitutotumori.mi.it (C.C.); 2 Laboratory of Structural Biology, Department of Biology, University of Rome “Tor Vergata”, 00133 Rome, Italy; desideri@uniroma2.it (A.D.); federico.iacovelli@uniroma2.it (F.I.); 3 Nuclear Medicine, Fondazione IRCCS Istituto Nazionale dei Tumori, 20133 Milan, Italy; ettore.seregni@istitutotumori.mi.it; 4 Fondazione IRCCS Istituto Nazionale dei Tumori, 20133 Milan, Italy; silvana.canevari@istitutotumori.mi.it

**Keywords:** prostate cancer, PSMA, monoclonal antibody, scFv, imaging, PET, CAR-T, theranostic

## Abstract

Prostate cancer (PCa) is the second leading cause of cancer among men, and its diagnosis and adequate staging are fundamental. Among the biomarkers identified in recent years for PCa management, prostate-specific-membrane-antigen (PSMA), physiologically expressed at a low level on healthy prostate and in other normal tissues and highly overexpressed in PCa, represents a reliable marker ideal for imaging and therapy. The development of anti-PSMA antibodies, such as D2B, demonstrated slow clearance of intact antibodies compared with fragments resulting in low tumor-to-blood ratios; however, the modular structural and functional nature of antibodies allowed the generation of smaller fragments, such as scFvs. In this review of the anti-PSMA antibody fragment scFvD2B, we combined further characterization of its biomolecular and tissue cross-reactivity characteristics with a comprehensive summary of what has already been performed in preclinical models to evaluate imaging and therapeutic activities. A molecular dynamics study was performed, and ScFvD2B occupied a limited conformational space, characterized by low-energy conformational basins, confirming the high stability of the protein structure. In the cross-reactivity study, the weak/absent immunoreactivity in non-tumor tissues was comparable to the PSMA expression reported in the literature. Biodistribution studies and therapeutic treatments were conducted in different animal models obtained by subcutaneous or locoregional injection of PSMA-positive-versus-negative xenografts. The maximum tumor uptake was observed for ^123^I(SPECT), ^124^I(PET), and optical imaging, which avoids kidney accumulation (compared with radiometals) and leads to an optimal tumor-to-kidney and tumor-to-background ratios. Regarding its possible use in therapy, experimental data suggested a strong and specific antitumor activity, in vitro and in vivo, obtained using CAR-T or NK-92/CAR cells expressing scFvD2B. Based on presented/reviewed data, we consider that scFvD2B, due to its versatility and robustness, seems to: (i) overcome some problems observed in other studied scFvs, very often relatively unstable and prone to form aggregates; (ii) have sufficient tumor-to-background ratios for targeting and imaging PSMA-expressing cancer; (iii) significantly redirect immune killing cells to PSMA-positive tumors when inserted in second-generation CAR-T or NK-92/CAR cells. These data suggest that our product can be considered the right reagent to fill the gap that still exists in PCa diagnosis and treatment.

## 1. Background

Prostate cancer (PCa) is the second leading cause of cancer among men, with an estimated 191,930 new cases and 33,330 deaths in 2020 in the United States [1]. Currently, PCa treatment depends on the stage of the disease at initial diagnosis; thus, diagnosis of PCa and adequate staging are fundamental for clinical and patient care, and imaging plays a central role.

Conventional imaging techniques such as bone scintigraphy, computed tomography, ultrasound, and magnetic resonance are current backbones in diagnostic medicine; these techniques are largely restricted to providing anatomical and physiological information. To optimize the management of PCa patients, positron emission tomography (PET) and single-photon emission computed tomography (SPECT) with emerging radiopharmaceuticals and fluorescence may provide accurate staging of primary disease, the restaging of tumor recurrence, and detection of metastatic disease. PET/CT allows direct visualization of tumor-dependent metabolism or target expression combined with morphological information, potentially allowing for detecting and localizing small lesions with an elevated metabolic rate or high target expression. Different PET radiotracers have been used to evaluate PCa, including ^18^F-fluorodeoxyglucose (^18^F-FDG), which has limited sensitivity due to low ^18^F-FDG-uptake in most PCa tumors, and ^11^C- or ^18^F-choline, but their accuracy is limited for initial staging and localization of tumor lesions in early biochemical recurrence [2,3,4,5].

Targeted therapies for cancer are aimed at maximizing tumor kill and minimizing toxicity, and their development requires, on one side, the identification of good targets and, on the other, site-selective targeting molecules. On the target side, in the last twenty years, many efforts have been aimed at identifying proteins present in cancer cells but not in normal cells or that are more abundant in cancer cells, where they exert a role in proliferation. Along with PSA, several new biomarkers have been identified in recent years for PCa, and they include cell surface proteins, glycoproteins, receptors, enzymes, and peptides [6].

**Figure 1:**
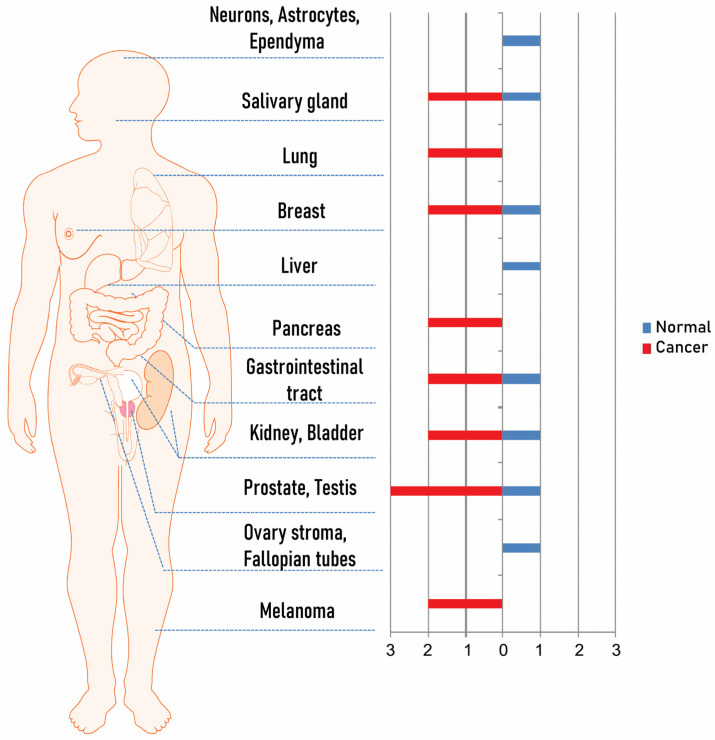
PSMA expression occurs in a range of solid tumors; the percentages of expression in tumor (red) and nonmalignant tissues (blue) have been derived from the literature using different detection techniques, numbers of specimens, and thresholds for determining expression [12,13,14,15,16,17].

Among biomarkers used for PCa managing, PSMA represents a valuable TAA (tumor-associated antigen) for PCa theranostics. PSMA is a rapid internalized and not-secreted receptor with folate hydrolase and carboxypeptidase activities. PSMA is a type II membrane glycoprotein (100–120 kDa) with an intracellular domain (amino acids 1–18), a transmembrane domain (amino acids 19–43), and an extracellular domain (amino acids 44–750) [7]. PSMA is physiologically expressed at a low level on healthy prostate and in other tissues, including, e.g., kidneys, gastrointestinal tract, brain, salivary glands, breast, kidney, and ovary [8,9]. In some of these organs, upon cancerization, PSMA expression significantly increases; in addition, a middle–high PSMA expression is recorded in other cancers rising from PSMA negative normal tissues such as lung and pancreas, and melanoma. PSMA levels increase up to 1000-fold on about 90% of primary and metastatic PCa cells including bone and lymph node metastases [8,9,10]. Most importantly, there is a strong correlation of PSMA expression with high tumor-grade and stage as well as metastatic and hormone-refractory disease [6,7,8,9,10,11]. Due to these properties, PSMA constitutes a reliable molecular marker for PCa and other PSMA-expressing tumors ideal for imaging and therapy. These data are summarized in Figure 1 [12,13,14,15,16,17].

On the side of selective targeting molecules, most of them are small molecules or monoclonal antibodies (mAbs). The latter represents one of the best examples of ‘intelligent’ bullets that arrive directly at the target with high target selectivity. Many mAbs are being tested or studied, and about thirty of them have already completed the experimental phase and have been introduced in the clinic [18]. In addition, the mAb molecular structure is easily manageable, revolutionizing the treatment of an ever-increasing number of cancers since they could be exploited as effective anticancer therapies using platforms such as antibody–drug conjugates, small-molecule drug conjugates, radioimmunoconjugates, bispecific antibodies, and chimeric antigen receptor T cells [19,20].

## 2. PSMA-Targeted Diagnosis/Therapy with Monoclonal Antibodies

The first commercialized anti-PSMA antibody was 7E11, which, as radioconjugate ^111^In-capromab-pendetide (ProstaScint) [21], was approved by the US Food and Drug Administration in 1996. However, ProstaScint binds to an intracellular epitope of PSMA and was therefore not capable of visualizing viable PCa cells, leading to poor clinical performance [22]. This limitation was partially overcome by the development of another anti-PSMA antibody, J591, that binds extracellular epitopes of PSMA, and was largely used in preclinical and clinical trials (ClinicalTrials.gov Identifier: NCT00195039; NCT02410577). Other mAbs directed to PSMA extracellular epitopes, as well as their conjugates, are being evaluated in a variety of experimental and preclinical models [23,24,25], but only IgGD2B [26], similarly to J591 [27], has been extensively characterized, demonstrating low tumor-to-blood ratios that can occur owing to relatively slow clearance of intact antibodies compared with fragments [28]. Different groups analyzed IgGD2B tumor-targeting capacity (Table 1, refs. [28,29,30,31,32] and compared it with those of F(Ab)_2_ and FAb fragments [28].

## 3. ScFvD2B Characterization and Development

Since the licensing of the first monoclonal antibody in 1986, antibodies have become the larger class of biopharmaceuticals [18]. However, the long circulatory half-life (3–4 days) typical of whole antibodies, as well as low tumor penetrability, the long delay between injection and imaging, and nonspecific accumulation associated with inflammation [33,34,35], limits their use in clinical practice. Utilizing engineered antibody fragments may address these challenges, as size reduction and removal of Fc function decrease serum half-life [28].

The modular structural and functional nature of Ab allowed the generation of smaller fragments, such as scFvs. These Ab fragments over a conventional Ab have several advantages (faster clearance from the circulation and shorter time for maximum tumor uptake) and some limitations (relative instability, low affinity [36], and absence of effector functions). The main history, format, and production methods are excellently summarized in a recent review [37]. IgGD2B antibody produced by conventional hybridoma technology was reshaped to scFv format, as described in Frigerio et al., 2013 [26]. ScFvD2B as its parental IgGD2B carries a V segment of the VK1 family (12/13 subgroup) and the VH belonging to the VH3 family.

ScFvD2B was initially produced in a prokaryotic system and later, in the roadmap for characterization of a clinical-grade reagent [38], in a GLP eukaryotic system. Our biochemical data indicate that scFvD2B did not aggregate, and not more than 5–8% dimers are present.

The binding specificity of our scFvD2B against PSMA was verified on different cell lines expressing or not expressing PSMA and showed good and specific binding to the cell lines expressing the antigen, whereas the non-related cell lines were negative. To enforce our data and add further characterization of scFvD2B in this current review, we also report some new and unpublished data.

**Figure 2:**
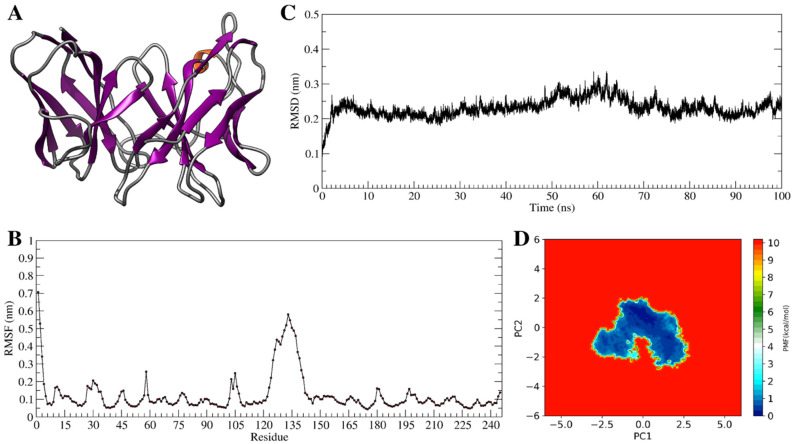
(**A**) ScFvD2B molecular model, built using the crystal structure of the single-chain variable fragment recognizing the human adiponectin receptor 2 (PDB ID: 5LX9, 2.4 Å resolution) from Mus musculus as a template [39]. The two sequences’ alignment shows a 60% identity. (**B**) Time-dependent root mean square deviation (RMSD) of the ScFvD2B model calculated for a 100 ns Gaussian Accelerated Molecular Dynamics (GAMD) simulation [40]. (**C**) The root mean square fluctuation (RMSF) values, describing the time-averaged deviation of the Cα atoms position, confirms the stability and the low fluctuation of the ScFvD2B structure. (**D**) Principal component analysis (PCA) analysis, coupled to the projection of the first two main motions to the reweighting of the GaMD simulation, recovering the original free energy profiles of the antibody fragment. The conformational space sampled by the single chain is directly proportional to the number of points plotted on the graph, whereas the color, ranging from blue to red, indicates the energy associated with the sampled conformations.

Since stability is a very important characteristic for a bioreagent, to further identify the scFvD2B physical property, a molecular dynamics simulation study was performed, as depicted in Figure 2. The root mean square deviation (RMSD) values, describing the deviation of the Cα atoms from the initial model as a function of time, indicate high stability of the protein structure. The largest flexibility was localized on the linker peptide connecting the heavy (VH) and light chains (VL). ScFvD2B model sampled a limited conformational space, characterized by low-energy basins, confirming its high stability.

These data suggest that our product shows to overcome some problems often observed in other studied scFvs, such as a relatively large instability [41] and a propensity to form aggregates [42,43]. Aggregation strongly can limit in vivo applications of protein therapeutics, including MAbs, since they may reduce the MAb efficacy and heavily contribute to immunogenicity.

His and Myc tag were also removed from ScFvD2B for a useful clinical-grade reagent with no change in biodistribution and targeting properties (data not shown).

In the perspective of clinical application, scFvD2B was evaluated by tissue cross-reactivity studies to assess possible reactions with non-target organs and tissues in humans. The study was carried out in accordance with the ‘Points to Consider in the Manufacture and Testing of Monoclonal Antibody Products for Human Use’ (1997) issued by the Centre for Biologics Evaluation and Research of the FDA, the ‘Guideline on Development, Production, Characterization and Specifications for Monoclonal Antibodies and Related Products’ (2008) issued by the EMEA/EMA and the EMA ICH S6 (R1) ‘Preclinical Safety Evaluation of Biotechnology-Derived Pharmaceuticals’ (2011) and subsequent addendum (2012). Specific binding of scFvD2B was weakly seen in epithelium of the prostate, breast, and pituitary, in the endothelium of the ovary and endometrium, and diffusely throughout myelinated regions of the cerebellum, cerebral cortex, spinal cord, colon, stomach, and peripheral nerve. These results are consistent with PSMA expression reported with anti-PSMA entire antibodies (see Figure 1). The weak binding to these non-tumor tissues is consistent with its expression and function since PSMA is responsible for folate transport and absorption in several tissues justifying its distribution. A similar immunohistochemical reactivity pattern on human frozen tissues was obtained with the anti-PSMA whole antibodies 7E11 and J591 without unacceptable side effects in prostate cancer patients.

## 4. ScfvD2B as a Diagnostic Agent

ScfvD2B can be exploited as an imaging diagnostic agent after conjugation with a label; chemical conjugation is a critical step in attaching isotopes to an antibody fragment. Radionuclide conjugation can be directly performed to the antibody fragment at certain amino acids or, for radiometals, indirectly performed through a chelating moiety, such as NOTA or DOTA. Labeling should proceed quickly, with high efficiency, and the immunoconjugate should be stable under in vivo conditions. Both direct conjugations may lead to the incorporation of the radionuclide at random sites on the antibody fragment, potentially impacting immunoreactivity if the antigen-binding site is modified and affecting biodistribution and pharmacokinetics of the probe. In fact, several studies [26,28] have demonstrated that many factors, such as the labeling chemistry, could influence the targeting and pharmacokinetics of an antibody fragment [44,45].

When proteins labeled with a radiometal bind to a rapid internalizing antigen such as PSMA, the label will be trapped in the lysosomes, leading to increased accumulation of activity over time. On the other hand, directly iodinated proteins will be rapidly catabolized and expelled from cells, reducing the kidney background noise. The rate of internalization is an intrinsic characteristic of the receptor and, upon binding with IgGD2B or its scFv, PSMA rapidly internalizes [26]; efficient receptor-mediated endocytosis should be considered as part of a therapeutic strategy through the use of engineered antibody conjugates. In addition to nuclear imaging, the use of antibodies and/or antibody fragments is increasingly being explored in the field of biomedical optical imaging, which mainly includes fluorescence or bioluminescence-based imaging probes. Optical imaging modalities have emerged as a preferable alternative over radioisotope-based imaging and have become increasingly popular for intraoperative staging to enable cellular resolution imaging of tumor margins during surgical resection. The main advantages are the high sensitivity, specificity, low cost, more practical fluorochrome design and conjugation method, portability of imaging instruments, and absence of ionizing radiations [46]. In near-infrared fluorescence (NIRF) optical imaging, cells are labeled with dyes or proteins that emit light of a limited spectrum when excited by different wavelengths of light.

ScFvD2B was conjugated with ^111^In and ^177^Lu, upon cross-ligation with NOTA and DOTA, respectively, and directly with the radio-iodinated isotopes ^123^I, ^124^I, and ^131^I and the fluorescent probe X770. The possibility to expose scFvD2B to harsh conditions, such as low pH during radiolabeling, is an important screening parameter for the selection of reagents suitable for diagnostic and therapeutic applications that require chemical manipulations without loss of specificity and functionality.

Biodistribution studies were conducted in different animal models obtained by subcutaneous or locoregional injection of PSMA-positive or PSMA-negative cell lines. In each model, tumor growth was monitored over time, and when the tumors reached an appropriate volume (200–400 mm^3^), non-tumor-bearing mice or mice bearing tumors in one or both flanks were injected via the tail vein with scFvD2B labeled. Despite scFvD2B’s rapid clearance from the blood, its strength of binding to PSMA was proved to be sufficient to have a rapid localization in target tissues. The maximum tumor uptake was observed for ^123^I (SPECT), ^124^I (PET), and optical imaging, which avoids kidney accumulation (compared with radiometals) and leads to an optimal tumor-to-kidney and tumor-to-background ratios.

**Figure 3:**
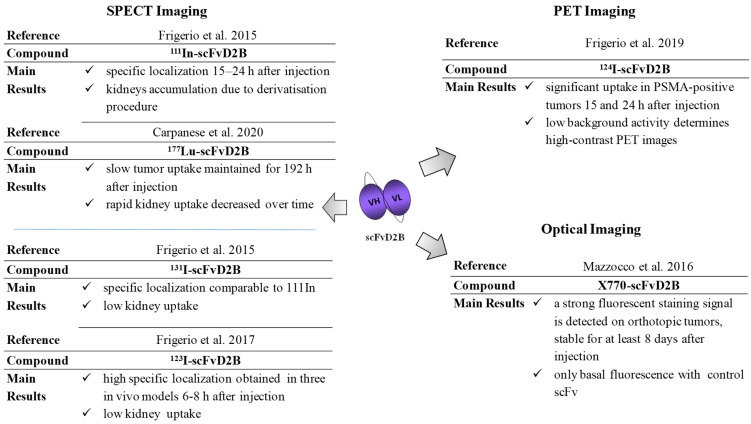
Summary of biodistribution and localization data with scFvD2B conjugates in PCa mice models.

The results of biodistribution obtained with the scFvD2B immunoconjugated with ^111^In, ^131^I, ^123^I, ^124^I, ^177^Lu, and X770 were summarized in Figure 3 [46,47,48,49,50].

In particular, the ability of X770-scFvD2B to detect prostate cancer was monitored by fluorescent reflectance imaging (FRI) and by fluorescence molecular tomography (FMT), and the accumulation of the labeled scFvD2B with respect to the labeled control scFv increased 10-fold at 72 h and 20-fold at 96 h.

## 5. ScFv as a Therapeutic Agent

Adoptive immunotherapy appears to be a promising strategy to control the advanced stages of cancer by specific targeting, in particular through chimeric antigen receptor T (CAR T) cell therapy or, more recently, CAR-NK [51,52,53]. The high curative potential against hematologic malignancies afforded by CAR T cells supports the large number of current clinical trials (nine directed against PSMA: 8 as CAR-T and 1 as CAR-NK) (www.Clinicaltrials.gov at 9 December 2021). Despite the advancements of CAR-T technology in the treatment of hematological malignancies, solid tumors still represent a challenge. To overcome current limits with CAR-T therapy, other cellular effectors than T lymphocytes are under study as possible candidates for CAR-engineered cancer immunotherapy. Nonetheless, CAR-T cell treatment is associated with heavy adverse events in a significant number of patients, and, additionally, the logistics and costs of such an approach pose significant challenges for making it available to a large number of potentially suitable recipients. A novel approach involves the NK-92 cell line, which mediates strong cytotoxic responses against various tumor cells but has no effect on nonmalignant healthy counterparts. Due to its versatility, scFvD2B was proposed as a therapeutic agent as second-generation CAR-T [54] or NK-92/CAR- to redirect immune killing cells on PSMA-positive tumors.

**Figure 4:**
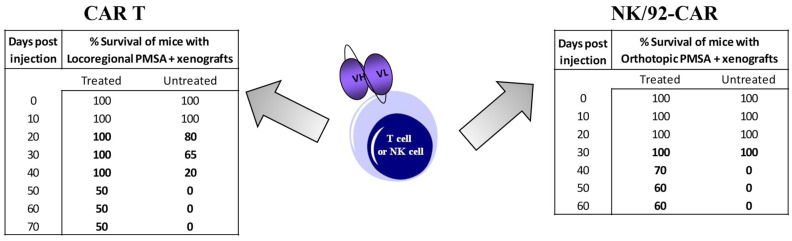
Antitumor activities over time of both scfvD2B CART (left panel) and NK/92 CAR (right panel), expressed as % of mice survival.

Zuccolotto et al. [55] demonstrated a strong and specific antitumor activity with scFvD2B CAR T, both in vitro and in vivo; in particular, they demonstrated that CAR-expressing lentiviral vectors efficiently transduced short-term activated PBMC, which, in turn, were readily stimulated to produce cytokines and to exert a relevant cytotoxic activity by engagement with PSMA+ prostate tumor cells. Upon in vivo transfer in tumor-bearing mice, CAR-transduced T cells were capable of completely eradicating disseminated neoplasia in more than 60% of treated NOD/SCID animals. Montagner et al. [56] demonstrated that the genetically modified NK-92/CAR cells acquired the ability to specifically and effectively lyse PSMA-expressing PCa cells, in contrast to parental NK/92 cells. Furthermore, the adoptive transfer of gene-modified NK-92 cells reduced tumor growth in different PCa mouse tumor models and significantly enhanced survival. Antitumor activities of both CAR-T and NK/92 CAR are shown in Figure 4 [55,56].

The robust ex vivo expansion of NK-92 cells to high numbers, the potent antitumor activity, the immediate availability as a fully defined and characterized cell product with reduced costs, and the lack of risks of manufacturing failures make this cell line an ideal platform for the development of off-the-shelf therapeutics CAR-engineered variants to target other solid tumors.

The therapeutic potential of scFvD2B, due to its advantageous properties and versatilities, could be exploited to redirect cytotoxic drugs such as immunotoxins [57] (currently under evaluation).

## 6. Conclusions

Based on the presented/reviewed data, we consider that the scFvD2B, due to its versatility and robustness, seems to: (i) overcome some problems observed with other scFvs, very often relatively unstable and prone to form aggregates; (ii) have sufficient tumor-to-background ratios for targeting and imaging PSMA-expressing cancer; (iii) significantly redirect immune killing cells to PSMA-positive tumors when inserted in second-generation CAR-T or NK-92/CAR cells. These data suggest that our product can be considered the right reagent to fill the gap in PCa diagnosis and treatment.

## Figures and Tables

**Table 1:** IgGD2B applications in PCa preclinical models [28,29,30,31,32].

Purpose	Imaging	Imaging/Therapy	Therapy	Therapy
Compound name	^111^In-IgGD2B	^111^In-DTPA-D2B-IRDye700DX	D2B–cisplatin–CNH conjugate	^223^RaA-silane-PEG-D2B
IgGD2B manipulation	Conjugation with ITC-DTPA and ^111^In radiolabeling	Conjugation with IRDye700DX and DTPA and ^111^In radiolabeling	Conjugation with CNH and cisplatin in a prodrug form	Conjugation with NaA zeolite nanocarrier and ^223^Ra radiolabeling
Model system	In vivo xenografts (PSMA+ vs. PSMA−	In vivo xenografts (PSMA+ vs. PSMA−)	In vitro cell lines (PSMA+ vs. PSMA−)	In vitro cell lines (PSMA+ vs. PSMA−)
Results	Xenografts clearly visualized in SPECT/CT with specific differentiation of PSMA+ imaging at 168 h post-injection	Xenografts clearly visualized using µSPECT/CT. PSMA-tPDT efficiently inhibit growth of PSMA-expressing tumors and prolong median survival	Selective binding, uptake, and killing of PSMA+ activities are enhanced when the nanosystems are shielded with BSA	Selective binding, uptake, and killing of PSMA+
Main Conclusions	Intact IgGD2B can be used when high concentrations of the antibody in the tumor are required	Near-infrared imaging can be used to guide surgical removal. PSMA-targeted photodynamic therapy (tPDT) can act on tumor remnants not removable surgically	This new system allows the variation of the quantity of drug or antibody attached to the nanostructure to play with the killing efficacy	^223^RaA-silane-PEG-D2B might be a promising agent for the targeted treatment of human PCa
Reference	[28,29]	[30]	[31]	[32]

## Data Availability

Not applicable.

## Abbreviations

CAR T	Chimeric Antigen Receptor T cell.
CT	computed tomography.
DOTA	1:4,7,10-tetraazacyclododecane-1,4,7,10-tetraacetic acid.
^18^F-FDG	^18^F-fluorodeoxyglucose.
FMT	fluorescence molecular tomography.
FRI	fluorescent reflectance imaging.
GAMD	Gaussian Accelerated Molecular Dynamics.
GLP	good laboratory practice.
Mab	monoclonal antibody.
NIRF	near-infrared fluorescence.
NOTA	2-(p-isothiocyanato benzyl)-1,4,7-triazacyclononane-1,4,7-triacetic acid.
NK	natural killer.
Pca	Prostate Cancer.
PCA	Principal component analysis.
PET	positron emission tomography.
PSMA	prostate-specific membrane antigen.
RMSD	root mean square deviation.
RMSF	root mean square fluctuation.
scFv	single-chain fragment variable.
SPECT	single-photon emission computed tomography.
TAA	tumor-associated antigen.
VH	heavy chain.
VL	light chains.
